# Unraveling the metabolic gene expression and energetic patterns of the seasonally acclimatized gilthead seabream

**DOI:** 10.1007/s10695-025-01513-y

**Published:** 2025-05-24

**Authors:** Vasiliki Makri, Ioannis A. Giantsis, Konstantinos Feidantsis, Ioannis Georgoulis, Antonia Gougousi, Basile Michaelidis

**Affiliations:** 1 Laboratory of Animal Physiology, Department of Zoology, School of Biology, Aristotle University of Thessaloniki, 54124 Thessaloniki, Greece; 2 Laboratory of Ichthyology & Fisheries, Faculty of Agriculture, Forestry and Natural Environment, Aristotle University of Thessaloniki, 54124 Thessaloniki, Greece; 3 Department of Fisheries & Aquaculture, School of Agricultural Sciences, University of Patras, 26504 Mesolonghi, Greece

**Keywords:** Farmed fish, Metabolic patterns, Seasonality, Lipid oxidation, Carbohydrate metabolism

## Abstract

The aim of the present study was to investigate how seasonal changes in the oxidation of biological energy substrates contribute to the thermal tolerance of farmed fish, as well as to explore the potential relationship between seasonality, metabolic pathways, and the energy reserves of a highly important aquaculture species, i.e., the gilthead sea bream *Sparus aurata*. In a monthly basis collected tissue samples from a fish farm in Evoikos Gulf in Greece, RNA/DNA ratio was measured, representing a highly informative index of the nutritional condition and growth of fish. Additionally, seasonal variations in glucose and lipid metabolism were assessed through relative gene expressions of key metabolic enzymes and proteins such as glucose transporter (Glu), lactate dehydrogenase (L-LDH), citrate synthase (CS), 3-hydroxyacyl-CoA dehydrogenase (HOAD), pyruvate kinase (PK), AMP-activated protein kinase (AMPK), and peroxisome proliferator-activated receptors (PPARα/γ). Furthermore, the expression of uncoupling proteins, NADH dehydrogenase (NDH-2), hypoxia-inducible factor-1 alpha (Hif-1a), electron transport system activity (ETS), and its components (complex I + III) was also employed as indicators of the respiratory chain activity. The findings reveal two distinct metabolic periods affecting productivity: a cold acclimatization phase marked by significant lipid accumulation and a warm acclimatization phase characterized by elevated carbohydrate metabolic pathways and enhanced corresponding enzymatic activities. However, the decreasing CS enzymatic activity during warm acclimatization may reflect the initiation of mitochondrial dysfunction. These metabolic adjustments underscore the fish adaptive responses to seasonal temperature fluctuations, highlighting their mechanisms of thermal tolerance and energy utilization. This understanding is particularly relevant for sustainability practices under varying thermal conditions.

## Introduction

One of the major critical requirements for an organism’s adaptation to alterations of environmental conditions and survival is the maintenance of energy balance, according to which energy input must meet the energy costs related to key physiological processes and functions such as activity, growth, development, and reproduction (Guderley and Pörtner 2010; Sokolova 2012, 2013). Energy production through oxidation of carbohydrates, fatty acids, and proteins recruits a variety of enzymes that are differentially affected by the changes of environmental conditions, among them temperature represents a primary factor influencing the rate of energy production (Hochachka and Somero 2002; Sébastien et al. 2021). To overcome the latter and compensate the effects of seasonal temperature fluctuations, fish recruit several adaptational mechanisms (e.g., behavioral, physiological and morphological adjustments). Specifically, the gilthead sea bream *Sparus aurata* (Linnaeus, 1758) has shown osmoregulatory and metabolic seasonal variations in experimental ponds (Vargas-Chacoff et al. 2009a), as well as seasonal variations in production of hormones regulated by temperature fluctuations (Vargas-Chacoff et al. 2009b) and similarly seasonal metabolic patterns in response to environmental changes such as seawater temperature (Vargas-Chacoff et al. 2009c). Moreover, metabolic reorganization and energy trade-offs between different tissues contribute to the maintenance of the physiological performance of organisms under stressful thermal conditions (St-Pierre et al. 1998; Pörtner et al. 2007; Guderley and Pörtner 2010; Kyprianou et al. 2010; Feidantsis et al. 2013, 2018; Makri et al. 2024).

Metabolic reorganization is regulated by a variety of cellular mechanisms, including gene expression and translational phenomena, which may be tissue specific (Guderley and Gawlicka 1992; Guderley 2004; Kassahn et al. 2009). However, several studies have shown that changes in mRNA expression for several proteins, including those related to enzymes involved in intermediary metabolism, are not always directly correlated with translational phenomena and changes in the corresponding protein levels and activity (Gracey and Cossins 2003; Logan and Somero 2011; Logan and Buckley 2015). The latter is characterized as a preparatory strategy which enables cells to defend further increases in the magnitude and duration of stressful conditions (Somero 2020). Integration of transcriptional, translational, and post-translational responses to abiotic stressors provides a more comprehensive understanding of organism—environment interactions on broader scales (Kassahn et al. 2009). Fish aquaculture systems can be characterized as a semi-wild habitat where individuals, although restricted into cages, are facing seasonal alteration of sea water temperature. Thus, understanding the physiological plasticity of fish to abiotic variables when acclimatized to field seasonal conditions will be important tools for evaluating the response of fish to the future oceanic conditions and extreme weather events associated with climate change (Pörtner and Farrell 2008; Somero 2010, 2020; Pörtner 2012; Bozinovic and Pörtner 2015).

In this context, and complementary to our previous work (Feidantsis et al. 2018, 2021), the present work aimed to further study the energetics and metabolic reorganization in the tissues of an important aquaculture species such as the gilthead sea bream *S. aurata* under field conditions. Since farmed fish in Mediterranean mariculture are exposed to both high and low temperatures, samples from different seasons were investigated at a monthly basis. The study is expected to lead to main outputs concerning the fish response depending on the season based on the trend in water temperature, as well as to practical implications regarding the organization of farm operations that should be considered to avoid losing growth potential, based on the development of nutritional approaches to mitigate the negative effects in each circumstance, i.e., increasing and decreasing water temperature. Specifically, the present study is focused on the metabolic and energy status of tissues, gene expression, and their protein products, correlated to carbohydrates, lipids metabolism, and respiratory chain. Energy status was based on the determination of RNA/DNA ratio, as a reliable indicator of general protein synthesis and metabolic activity, to assess fish nutritional condition (Dahlhoff 2004; Stevenson et al. 2006), and AMP-activated protein kinase (AMPK) as a sensor of energy status that maintains cellular energy homeostasis, while additionally, it is involved in the expression of several genes related to several metabolic pathways (Garcia and Shaw 2017). As reported elsewhere, oxygen consumption is temperature depending in *S. aurata* (Ibarz et al. 2010). Accordingly, we further investigated hypoxia induced factor (*hif*-1α) gene expression levels as an indicator of seasonal induced hypoxia. To assess carbohydrate metabolism, we determined the relative gene expression of glucose transporter (*glu*), pyruvate kinase (*pk*), L-lactate dehydrogenase (*ldh*), citrate synthase (*cs*), and the enzymatic activities of PK, CS and L-LDH, and blood plasma glucose levels. Likewise, for fatty acid oxidation, we investigated carnitine palmitoyl transferase (*cpt*) and 3-hydroxyacyl CoA dehydrogenase (*hoad*) gene expression, the enzymatic activity of HOAD, and blood plasma triglycerides levels. Moreover, and targeting on lipid metabolism, we examined peroxisome proliferator-activated receptor α/γ (*ppar α/γ*) and fatty acid binding proteins (*fabp2 a/b*) gene expression levels. In this sense, NADH dehydrogenase (*ndh*-2) and cytochrome c oxidase (*cox1*) gene expression and enzymatic activities were also determined, additionally to CI + CIII complex activity and uncoupling proteins (*ucp1*, *ucp2*, and *ucp3*) gene expression levels. Finally, and in order to provide data regarding energy production and utilization, we examined total ATP, adenylate energy charge (AEC), and the activity of electron transfer system (ETS) levels.

## Materials and methods

### Animals and tissue sampling

**Fig. 1:**
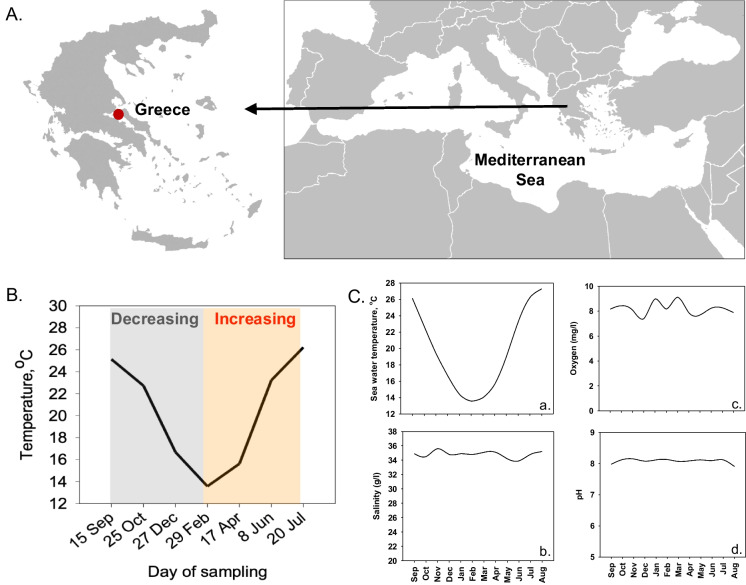
**A** Study area in North Evoikos Gulf, Greece. **B** Seasonal variations of sea water temperatures in the study area and during samplings. **C** Annual variations of (a) sea water temperatures, (b) salinity, (c) dissolved oxygen, and (d) pH

Adult farmed *S. aurata* individuals (mean ± SD weight of 545 ± 6.5 g, ~ 3 y.o.) were collected (*n* = 6) in a seasonal sampling scheme, at both decreasing and increasing ambient sea water temperature from an aquaculture unit located in Evoikos Gulf, Greece (Fig. 1A). Although reproductive maturation and spawning in *S. aurata* is set in the cold autumn—winter months (Feidantsis et al. 2013; Fateh et al. 2018), the individuals of the present study exhibited no obvious gonads, and thus, they were designated as reproductively immature females. The aforementioned size represents the market size of the sea bream that also corresponds to the point that the growth is stabilized reaching maturity. The good condition, health, and welfare of fish were confirmed from the aquaculture unit personnel who check at a daily basis a variety of indicators to prevent any harmful event or fish escape. Fish were fed daily until satiation with a commercial fish feed with the following proximate composition (ingredients (g kg^−1^)): fish meal 650, fish oil 90, wheat 159, wheat gluten 63, vitamin and mineral mix 25, DL-methionine 3, L-lysine 0, Celite 10, crude protein 573 ± 14, adjusted protein 562 (protein adjusted for the acid insoluble nitrogen), crude fat 131 ± 4, ash 111 ± 1, crude fiber 15 ± 1, acid detergent fiber 63 ± 3, energy 22.5 ± 0.1 MJ kg^−1^. The vitamin and mineral premix consisted of (kg^−1^): choline 90,000 (mg), vitamin A 0.3 (MIU), vitamin D3 0.1 (MIU), vitamin E20,000 (IU), vitamin K 1030 (mg), vitamin B1 390 (mg), vitamin B 960 (mg), nicotinic acid 2600 (mg), pantothenic acid 4400 (mg), vitamin B6 890 (mg), vitamin B12 15 (mg), folic acid 290 (mg), biotin 14 (mg), vitamin C (Stay C 35% MONO) 20,300 (mg), inositol 15,600 (mg), total Mn 1200 (mg), total Ca 72,000 (mg), total Zn 7000 (mg), total Cu 450 (mg), total Se 14 (mg), total I 100 (mg), betaine 71,250 (mg), BHA (E320) 3000 (mg). The seasonal profile of sea water temperature was divided in a cold acclimatization period (15 September 2020–29 February 2021) characterized by decreasing temperatures and a warm acclimatization period (29 February 2021–20 July 2021) characterized by increasing temperature (Fig. 1B). Sea water temperature variations were measured in the field using a Multiparameter Water Quality Meter (Model WQC-24, DKK-TOA Company). Parallel to the water temperature, the salinity, the concentration in oxygen, and the pH were also recorded at 12 a.m. and 12 p.m. daily in a monthly basis (Fig. 1C).

Fish samplings were seasonal and were carried out based on the pattern of seasonal temperature changes in the area in previous years. A total of 7 seasonal samplings were carried out with the first one in September and the last in July. Daily feed intake was similar between samplings, whereas all fish originated from the same cage. It should be also noted that since all fish were from the same hatchery that belongs to the same company, they had the same progeny, and thus identical genetic background. Immediately after collection, fish were placed in sea water containing MS-222 to a final concentration of 0.15 g L^–1^ for anesthesia. Thereafter, blood was sampled from the dorsal aorta with a heparinized syringe (Smith and Bell 1964) and was immediately frozen in liquid nitrogen. Then, fish were measured and dissected, and heart, liver, and white and red muscle samples were removed, frozen in liquid nitrogen, transported to the laboratory, and maintained at − 80 °C until further analytical processes. For every biochemical analytical process of this study, tissues from 6 individuals of each sampling (*n* = 6 biological replicates) were used. Specifically, from each individual, three technical replicates were obtained for each tissue and pooled to be further proceeded to the following analytical procedures that were performed in six replicates.

### Analytical procedures

#### RNA/DNA ratio

For the determination of RNA/DNA ratio, 0.2 g tissue was placed in RLT Plus buffer (Qiagen, Germany) and extraction of genomic DNA and total RNA was performed using the AllPrep DNA/RNA Mini Kit (Qiagen, Germany). Following this protocol, extraction of both nucleic acids was performed simultaneously by the same tissue sample, in an effort to eliminate the possibility of misleading results. Briefly, the homogenate was embedded in lysis buffer and passed through a first silica column that binds DNA and then through a second column which selectively binds RNA. Flow through the DNA column was eventually diluted in 50 µL of Qiagen elution buffer, whereas flow through of the RNA column was diluted in 50 µL ultrapure water and their concentrations were measured in a Quawell Q5000 micro-volume UV–Vis spectrophotometer (Quawell Technology, China).

#### Gene expression

Quantitative real-time PCR for estimation of gene expression profiles was performed on all four examined tissues (heart, liver, white and red muscle). Initially, RNA was extracted from homogenized tissues using the Nucleozol purification kit (Macherey–Nagel, Germany) following the manufacturer’s recommended protocol. Furthermore, 1 µL of extracted RNA of approximate concentration 100 ng µL^−1^ was subjected to first strand cDNA synthesis using the PrimeScript kit (Takara, Japan) applying the oligodT primers option. The expression levels of *glu*, *ldh*, *cs*, *cox*, *pk*, *ldh*, *hoad*, *ucp1*, *ucp2*, *ucp3*, *ndh-2*, *ampkγ2*, *hif-1a*, *ppar α/γ*, and *fabp2 a/b* were determined in a PCRmax Eco 48 instrument using the AMPLIFYME SYBR Universal Mix kit (blirt, Poland) in 10-µL reactions containing 5 µL 2 × AMPLIFYME ready for use, 0.3 pmol of each forward and reverse primer, 1 µL cDNA of approximate concentration 50 ng/µL, and ultrapure water up to the final volume of 10 µL. Primer pairs for each amplified gene are presented in Table 1. Reference genes for quantification purposes were the elongation factor (*ef-1*) and the ribosomal gene *l13a*. Reactions were run with an initial denaturation step for 3 min 95 °C, followed by 40 cycles of 20 s at 95 °C, 15 s at 60 °C, and 10 s at 72 °C. Relative quantification was calculated from the comparison of the investigated target genes cycle threshold values (*C*_T_) with the two mentioned genes 2^−ΔΔCt^ quantification methodology (Livak and Schmittgen 2001). In order to verify the validity and the stability of the reference genes, initially, the EF-1 and L13a genes were amplified in 10 random cDNA samples. The ΔCt values of these genes were inserted in the software Normfinder (Andersen et al. 2004) to estimate their stability index (Bustin et al. 2009). The expression level of each gene was then estimated by the 2^−ΔΔCt^ quantification method, exploring three replicates per sample with normalization being carried out using *ef-1* gene. No melt curve was conducted, but instead, PCR products were verified in terms of length in an agarose gel electrophoresis, whereas the efficiency of all genes analyzed was greater than 90% and lower than 110% (Table 1).

**Table 1:** Primer pairs used for the gene expression analysis

Gene targeted	Primer sequence (5′−3′)	Amplified product length	Primer efficiency	Reference
*glu*	F: GCTTGGTTGGATGCCTATGT R: AGGACTCTGTTGCCGCTTT	88 bp	95.6%	(NCBI Reference Sequence: XM030416074)*
*ndh-2*	F: CACCTGGAACAAGACCAATAAC R: TTCAAAGGGAGGAAGAGGAC	168 bp	95.8%	(NCBI Reference Sequence: XM_030421834.1)*
*cox1*	F: ACCCTGAGTCCAGAGCAGAAGTCC R: AGCCAGTGAAGCCGATGAGAAAGAAC	187 bp	97.1%	Bermejo-Nogales et al. (2014)
*ucp1*	F: ATGACGCAGCGTGTTTTCTG R: CCTGTTTTACATAGAGAGTTGCAC	217 bp	102.1%	(NCBI Reference Sequence: FJ710211.1)*
*ucp2*	F: ACAAGATATATGAACGCCCGCT R: TGCTGTCATCATGGCTCGTT	168 bp	96.2%	(NCBI Reference Sequence: XM_030438823.1)*
*ucp3*	F: GTGAGCAGTGGTTGGAAAGG R: TCCATCACACCCGTCGTTTA	190 bp	101.3%	(NCBI Reference Sequence: XM_030425767.1)*
*ldh*	F: ATCCCGAACATCATCGTCAAGTA R: TTGATAACCTCGTAGGCTCC	368 bp	96.3%	(NCBI Reference Sequence: XM_030425767.1)*
*cs*	F: TCCAGGAGGTGACGAGCC R: GTGACCAGCAGCCAGAAGAG	51 bp	95.8%	Bermejo-Nogales et al. 2014
*hoad*	F: TCACTTCTTCAACCCAGTCC R: GTTGACAATGAATCCCGGTG	154 bp	96.2%	(NCBI Reference Sequence: XM_030431227.1)*
*pk*	F: CGCCCAGAAGATGATGATTGG R: ATTACACAGTCGGCCCATC	156 bp	98%	(NCBI Reference Sequence: KF857579.1)*
*ampkγ2*	F: GACATCGCTTTCATCCACCC R: TACGTCTTCTCAGCAGCCA	158 bp	93.4%	(NCBI Reference Sequence: XR_003982169.1)*
*hif-1a*	F:AAACACAAGCCACTGTCATC R: AACTTCTCCTCCACCTCCTC	235 bp	94.1%	(NCBI Reference Sequence: XM_030443698.1)*
*cpt*	F: AACCTCATCAACTTCCACATC R: TCCAAATTCGTCTCAATCATCC	168 bp	97.8%	(NCBI Reference Sequence: XM_030426203.1)*
*pparα*	F: TTCGTGGCTGCCATTATCTG R: CACCAAAGGCACATCCACC	60 bp	98.3%	Leaver et al. (2005)
*pparγ*	F: GCCTCAATGTCGGCATGT R: TCCTTCTCCGCCTGGG	64 bp	97.5%	Leaver et al. (2005)
*fabp2a*	F: GCTGGCTGCTCACGACAAC R: CGTGATCAGTTTGGTCTAAGC	325 bp	91.2%	Kaitetzidou et al. (2015)
*fabp2b*	F: CCGCAACGACAACTATGATAAG R: TGGACTCTTTGATGTGAAACTTG	131 bp	96.6%	Kaitetzidou et al. (2015)
*ef-1*	F: CCCGCCTCTGTTGCCTTCG R: CAGCAGTGTGGTTCCGTTAGC	135 bp	98.8%	Bermejo-Nogales et al. (2014)
*l13a*	F: TCTGGAGGACTGTCAGGGGCATGC R: AGACGCACAATCTTAAGAGCAG	148 bp	94.8%	Kaitetzidou et al. (2015)

*Designed in this study

### Determination of AMPK levels

The preparation of tissue samples for SDS-PAGE and the immunoblot analysis are based on well-established protocols. In the present study, equivalent amounts of proteins (50 µg) were separated on 10% and 0.275% (w/v) acrylamide and bisacrylamide slab gels, respectively, and transferred electrophoretically onto nitrocellulose membranes (0.45 µm, Schleicher and Schuell, Keene N. H. 03431, USA). The antibodies used were monoclonal anti-AMPK (5831, Cell Signaling) and monoclonal anti-β-actin (3700, Cell Signaling). After washing in TBST (20 mM Tris–HCl, pH 7.5, 137 mM NaCl, 0.1% (v/v) Tween 20) (3 times, 5 min each), blots were incubated with horseradish peroxidase-linked secondary antibodies in 1% dried non-fat milk in TBST and washed again in TBST (3 times, 5 min each), and the blots were detected employing enhanced chemiluminescence (Chemicon) with exposure to Fuji Medical X-ray films. Films were quantified by laser-scanning densitometry (GelPro Analyzer Software, GraphPad, USA).

### Determination of enzyme activities in the tissue homogenates

Preparation of homogenates for assaying L-lactate dehydrogenase (L-LDH, EC 1.1.1.27), pyruvate kinase (PK, EC 2.7.1.40), citrate synthase (CS, EC 4.1.3.7), and 3-hydroxyacyl CoA dehydrogenase (HOAD, EC1.1.1.35) was based on techniques by Driedzic and Almeida-Val (1996). NADH dehydrogenase (NDH-2, EC 1.6.5.3), cytochrome c oxidase (COX, EC 1.9.3.1), and complex CI + CIII homogenates were prepared as described in Hunter-Manseau et al. (2019). Specifically, for the analysis of LDH, PK, and HOAD activities, samples were homogenized in a buffer containing 150 mM imidazole, 1 mM EDTA, 5 mM dithiothreitol (DTT), and 1% Triton X-100, pH 7.4. For CS activity, tissue samples were homogenized in a buffer containing 20 mM HEPES, 1 mM EDTA, with 1% Triton X-100, and pH 7.4. For the analysis of NDH-2, COX, and CI + CIII activities, samples were homogenized in a buffer containing 50 mM imidazole-HCI, 2 mM MgCl_2_, 5 mM ethylene diamine tetra acetic acid (EDTA), 1 mM reduced glutathione, and 0.2 mM fructose-2,6-bisphosphate, pH 7.5. To avoid loss of enzyme activity during sample preparation, procedures were performed on ice. Before analysis, homogenates were centrifuged at 13,000 × *g* for 10 min at 4 °C and spectrophotometry was applied in the aqueous layer.

The enzymatic activities (*V*_max_) were determined spectrophotometrically, and all assays were based on well-established protocols for fish tissues (Moon and Mommsen,^1987^; Singer and Ballantyne 1989; Hunter-Manseau et al. 2019).

PK activity was measured at 340 nm by following the oxidation of NADH (extinction coefficient *ε*_340_ = 6.22 ml cm^–1^ µmol^–1^) in a medium containing 50 mM imidazole–HCl, 10 mM MgCl_2_, 100 mM KCl, 5 mM ADP, 0.15 mM NADH, 5 mM phosphoenolpyruvate, 0.6 U ml^–1^ lactate dehydrogenase, and pH 7.4 (Moon and Mommsen 1987).

L-LDH activity was measured at 340 nm by following the oxidation of NADH (extinction coefficient *ε*_340_ = 6.22 ml cm^–1^ µmol^–1^). *V*_max_ was determined in a medium containing 0.15 mmol L^–1^ NADH, 1 mmol L^–1^ KCN, 50 mmol L^–1^ imidazole, and pH 7.4. The reaction was initiated by adding 1 mmol L^−1^ pyruvate (Moon and Mommsen 1987).

HOAD activity was measured at 340 nm by following the oxidation of NADH (extinction coefficient *ε*_340_ = 6.22 ml cm^–1^ µmol^–1^). *V*_max_ was determined in a medium containing 0.15 mmol L^–1^ NADH, 1 mmol L^–1^ KCN, 1 mmol L^–1^ EDTA, 50 mmol L^–1^ Imidazole, and pH 7.4. The reaction was initiated by the addition of 2.0 mmol L^–1^ acetoacetyl CoA (Moon and Mommsen 1987).

CS activity was measured at 412 nm, following the reduction of 5,5-dithiobis-2-nitrobenzoic acid (DTNB, extinction coefficient *ε*_412_ = 14.15 ml cm^−1^ mmol^−1^) using a reaction medium containing 100 mM imidazole–HCl, 0.1 mM DTNB, 0.1 mM acetyl-CoA, and 0.15 m M oxaloacetic acid, pH 8.0 (Singer and Ballantyne 1989).

NDH−2 activity was measured at 600 nm to follow the reduction of 2,6-dichloroindophenol (DCIP, extinction coefficient *ε*_600_ = 19.1 ml cm^–1^ µmol^–1^) in a medium containing 100 mM imidazole, 2.5 mg ml^–1^ BSA, 5 mM MgCl_2_, 4 µM antimycin A, 10 mM sodium azide, 50 µM DCIP, 65 µM (ubiquinone coenzyme Q1), and pH 8.0. The reaction was initiated by addition of 0.14 mM NADH (Hunter-Manseau et al. 2019).

COX1 activity was measured at 550 nm, monitoring the oxidation of reduced cytochrome c (extinction coefficient *ε*_550_ = 29.5 ml cm^−1^ mmol^−1^) in a reaction medium containing 100 mM potassium phosphate, 0.05% (v/v) Tween 20- and 100-mM equine heart cytochrome c, and pH 8.0 (Hunter-Manseau et al. 2019).

Complex CI + CIII activity was measured at 490 nm following reduction of p-iodonitrotetrazolium violet (INT, extinction coefficient *ε*_490_ = 15.91 ml cm^–1^ µmol^–1^) in a medium containing 100 mM potassium phosphate, 0.85 mM NADH, 2 mM INT, 0.2% (v/v) Triton X-100, and pH 8.5 (Hunter-Manseau et al. 2019).

### Determination of ETS

ETS was determined by adding NADPH solution (300 µL) in tissue homogenate (10 mg) and INT (p-iodonitrotetrazolium, Sigma-Aldrich), following the increase in absorbance at 490 nm for 3 min (Haider et al. 2017). The calculation of ETS is based on the assumption that the equation of 1 µmole formazan corresponds to 0.5 µmole O_2_ (Gnaiger 1983; Haider et al. 2017).

### Metabolites analysis (ATP and AEC)

Perchloric acid (PCA, 1:7 w/v) was added in homogenized samples, and then, samples were centrifuged (10,000 rpm, 10 min, 4 °C). In the supernatant, an appropriate volume of KHCO_3_ was added in order to achieve pH 7. Then, samples were again centrifuged (14,000 rpm, 10 min, 4 °C). ATP, ADP and AMP were spectrophotometrically determined at 340 nm (Adam 1963). Adenylate energy charge was calculated by mathematic formula: AEC = ([ATP] + 0.5 [ADP])/([ATP] + [ADP] + [AMP]).

### Determination of glucose and triglycerides in blood plasma

Glucose and triglycerides were determined in plasma sampled from individual fish in March, May, and August, using commercial kits based on enzymatic–colorimetric methods from Spinreact, Spain (glucose kit, cod. 1,001,191; triglycerides kit, cod. 1,001,312).

### Statistics

The statistical analysis of results was performed using SPSS 22.0. Comparisons among samples were made by one-way analysis of variance (ANOVA), attributing significance to 5% confidence level (*p* < 0.05). Post-hoc comparisons were performed using Tukey–Kramer post-test. Friedman’s non-parametric test, followed by Dunn’s post-test, was performed to re-analyze and cross-examine our data. Values are presented as means ± S.D. Principal component analysis (PCA) was conducted using the FactoMineR package in R (Lê et al. 2008) in order to determine patterns of correlated variables.

## Results

### Sea water physicochemical parameters

Figure 1B illustrates the ambient sea water temperature recorded at a monthly basis. The lowest temperature, 12.86 °C, was recorded in February, while the highest temperature, 28 °C, was observed in both July and August. The data were categorized into two periods based on temperature: the first period, from September to February, was characterized by low (decreasing) temperatures, while the second period, from February to July, was marked by higher (increasing) temperatures. Figure 1C illustrates the annual cycle of the levels of sea water temperature, dissolved oxygen, salinity, and pH. Compared to the sea water temperature, the levels of dissolved oxygen, salinity, and pH remain unchanged throughout the year.

### RNA/DNA, Hif1-α, and AMPK

**Fig. 2:**
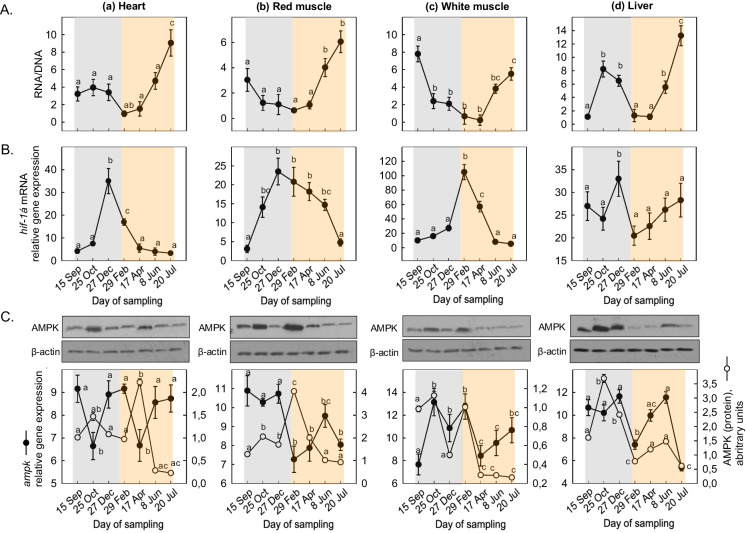
Seasonal changes in **A** RNA/DNA ratio, **B** *hif-1α* gene expression, and **C** AMPK gene expression and protein levels (against β-actin) in the (a) heart, (b) red muscle, (c) white muscle, and (d) liver of *Sparus aurata* (*n* = 6 preparations from different animals, values are presented as means ± S.D.). Light grey and light orange rectangles depict decreasing and increasing sea water temperature, respectively. Representative blots are shown. Statistically significant differences (*p* < 0.05) between different samplings are indicated by lowercase letters

RNA/DNA ratio was used as a marker of the nutritional condition of fish. In the heart, RNA/DNA ratio decreased from September until late in February, followed by a sharp increase from April to mid-July (Fig. 2Aa). In the red muscle, RNA/DNA ratio gradually declined late in December, followed by a significant sharp increase from April to July (Fig. 2Ab). In the white muscle, the ratio showed a sharp decrease from September to October and then gradually decreased until February, followed by a notable increase from April to July (Fig. 2Ac). In the liver, RNA/DNA ratio exhibited a rapid increase from September until late in October, followed by a significant increase from April to July (Fig. 2Ad).

*hif*-α mRNA in the heart and red and white muscle exhibited increasing levels from September to February and thereafter depicted decreasing levels (Fig. 2Ba, Bb, Bc). In the liver, while *hif*-α mRNA levels exhibited a similar pattern of expression compared to the other tissues in the period from September to February, but during the decreasing ambient sea water temperature, they sharply decreased exhibiting the lowest levels in February and thereafter gradually increased until June (Fig. 2Bd).

*ampk* mRNA levels in the heart showed two sharp declines: the first one from September to December and the second one from February to April. On the contrary, AMPK protein levels exhibited a significant peak in October, followed by a decrease compared to the initial protein levels (Fig. 2Ca). In the red muscle, *ampk* mRNA levels decreased from December to February, followed by an increase until June. However, AMPK protein levels in the red muscle peaked from February to July (Fig. 2Cb). In the white muscle, *ampk* mRNA levels showed two significant peaks, one in October and another one in February, followed by a gradual increase until July. The AMPK protein levels displayed a pattern similar to that of the gene expression (Fig. 2Cc). In the liver, AMPK mRNA levels showed a sharp decline from December to February, followed by an increase in June, and then a decrease in July. AMPK protein levels in the liver increased significantly from September to October, followed by a gradual decline from October until April (Fig. 2Cd).

### Indices of carbohydrate metabolism

**Fig. 3:**
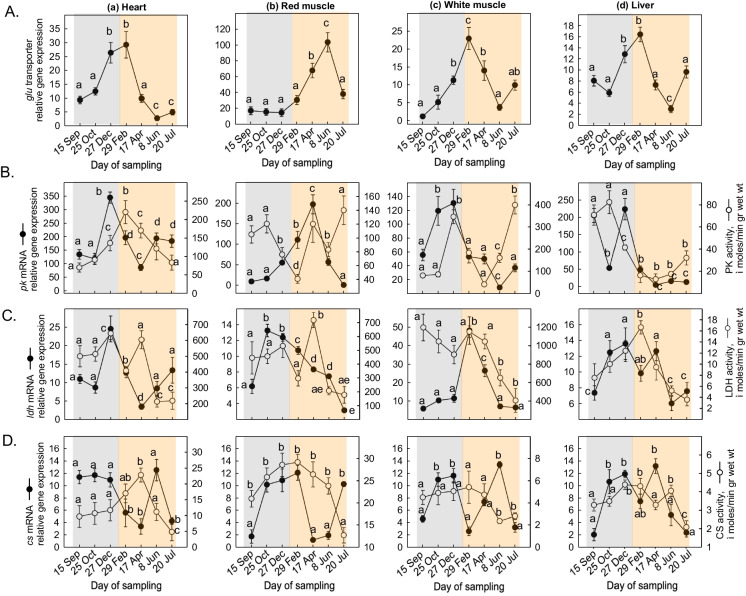
Seasonal changes in **A** *glu* transporter gene expression, **B** PK, **C** L-LDH, and **D** CS gene expression and enzymatic activity levels in the (a) heart, (b) red muscle, (c) white muscle and (d) liver of *Sparus aurata* (*n* = 6 preparations from different animals, values are presented as means ± S.D.). Light grey and light orange rectangles depict decreasing and increasing sea water temperature, respectively. Statistically significant differences (*p* < 0.05) between different samplings are indicated by lowercase letters

*glu* mRNA levels significantly increased in the heart from September to February, followed by a gradual decrease until June (Fig. 3Aa). In the red muscle, *glu* mRNA levels showed a stability from September to December (Fig. 3Ab), while in the white muscle, relative gene expression levels sharply increased from September to July (Fig. 3Ac). In the liver, *glu* mRNA levels rapidly increased from October to December and then declined from April to July (Fig. 3Ad).

*pk* mRNA levels exhibited high levels of expression in the heart from September to December. PK enzymatic activity levels showed a gradual increase from September to February, followed by a decrease until July (Fig. 3Ba). In the red muscle, *pk* mRNA levels gradually increased from September to April, while PK enzymatic activity levels increased from December to February, followed by a sharp decrease in June (Fig. 3Bb). In the white muscle, *pk* mRNA levels showed a significant increase from September to December and then gradually decreased until June. PK enzymatic activity levels exhibited two significant peaks: one in December and one in July (Fig. 3Bc). In the liver, *pk* mRNA levels sharply decreased from September to October, significantly increased in December, and then decreased from April to June. PK enzymatic activity levels in the liver gradually decreased from October to February (Fig. 3Bd).

*ldh* mRNA levels increased in the heart from October until late in December, followed by a decrease until mid-July. L-LDH enzymatic activity levels sharply increased in April and then gradually decreased until July (Fig. 3Ca). In the red muscle, *ldh* gene expression increased from September to February, followed by a gradual decrease in mRNA levels until July. However, L-LDH enzymatic activity levels in the red muscle showed a decline from April to July (Fig. 3Cb). In the white muscle, *ldh* mRNA levels exhibited a significant peak in February, but enzymatic activity levels gradually declined from February to July (Fig. 3Cc). In the liver, *ldh *mRNA levels showed a slight gradual increase from September to December, followed by decreased levels from mid-June to July. L-LDH enzymatic activity levels in the liver gradually increased from September to February and then decreased and remained low until July (Fig. 3Cd).

*cs* mRNA levels in the heart demonstrated a gradual decline from September to April, followed by a significant increase in June. CS enzymatic activity levels depicted a significant gradual decrease from September to April, followed by an increase until July (Fig. 3Da). In the red muscle, *cs* mRNA levels sharply increased from September to February, then significantly decreased in April, and recovered again to high levels in July. CS enzymatic activity levels in the red muscle progressively increased from September to July (Fig. 3Db). In the white muscle, *cs* gene expression increased from September to December, followed by a gradual increase in June. *cs* mRNA levels in the white muscle decreased from April to June (Fig. 3Dc). In the liver, *cs* mRNA levels exhibited high levels in December and a significant peak in April, but CS enzymatic activity levels displayed an opposite pattern of activity (Fig. 3Dd).

### Indices of beta-oxidation and transporters of lipids

**Fig. 4:**
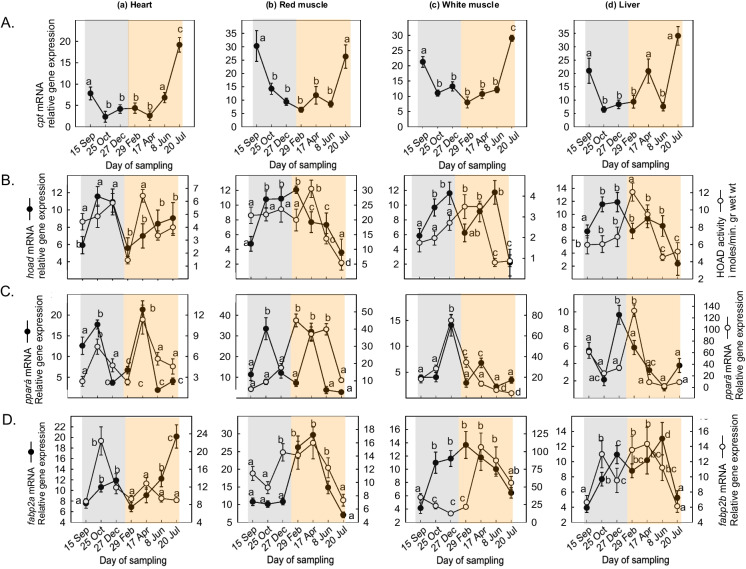
Seasonal changes in **A** *cpt *gene expression, **B** HOAD gene expression and enzymatic activity, **C** *ppar *α/γ, and **D** *fabp*2 a/b gene expression levels in the (a) heart, (b) red muscle, (c) white muscle, and (d) liver of *Sparus aurata* (*n* = 6 preparations from different animals, values are presented as means ± S.D.). Light grey and light orange rectangles depict decreasing and increasing sea water temperature, respectively. Statistically significant differences (*p* < 0.05) between different samplings are indicated by lowercase letters

*cpt* mRNA levels in the heart significantly increased in July (Fig. 4Aa). In the red muscle, *cpt* mRNA levels showed a sharp decline from September to October, remained stable, and then increased significantly in July (Fig. 4Ab). A similar pattern was observed in the white muscle (Fig. 4Ac) and in the liver (Fig. 4Ad).

*hoad* mRNA levels increased in the heart from September to October and remained stable during the other months. HOAD enzymatic activity levels depicted a significant increase from September to April, returning to initial levels in June (Fig. 4Ba). In the red muscle, *hoad* mRNA levels significantly increased from September to February and then gradually decreased until July. In contrast, HOAD enzymatic activity levels did not show significant changes (Fig. 4Bb). In the white muscle, *hoad* mRNA levels increased from September to December, while enzymatic activity levels increased from April to July (Fig. 4Bc). In the liver, *hoad* mRNA levels increased slightly from September to October, while the enzymatic activity levels gradually increased from September to February, followed by decreased values until July (Fig. 4Bd).

*ppar*α mRNA levels peaked significantly in the heart in April (Fig. 4Ca). In the red muscle, *ppar*α mRNA levels recorded two peaks, one in October and another one in April (Fig. 4Cb). In the white muscle, *ppar*α mRNA levels sharply increased from September to December and thereafter returned to initial levels (Fig. 4Cc). In the liver, a sharp increase was observed from October to February, followed by a gradual decrease in mRNA levels until June (Fig. 4Cd). *ppar*γ mRNA levels rapidly increased in the heart from February to April (Fig. 4Ca). In the red muscle, *ppar*γ mRNA levels progressively increased from September to February and then returned to initial levels until July (Fig. 4Cb). In the white muscle, a sharp increase in mRNA levels was evidenced from September to December, followed by a gradual decline until July (Fig. 4Cc). In the liver, *ppar*γ mRNA levels exhibited a sharp increase from December to February (Fig. 4Cd).

*fabp*2a mRNA levels significantly increased in the heart from December to February, followed by a gradual decrease until July (Fig. 4Da). In the red muscle, *fabp*2a mRNA levels increased in April, followed by a decrease until July (Fig. 4Db). In the white muscle, *fabp*2a mRNA levels increased from September to October and then decreased from February to July (Fig. 4Dc). In the liver, *fabp*2a mRNA levels slightly increased from October to February and remained stable (Fig. 4Dd). Conversely, *fabp*2b mRNA levels sharply increased in the heart from December to February and returned to initial levels by July (Fig. 4Da). A similar pattern of expression was observed in the red muscle (Fig. 4Db). In the white muscle, *fabp*2b mRNA levels increased slightly from October to December (Fig. 4Dc), while in the liver, no significant changes were observed (Fig. 4Dd).

### Mitochondrial enzymes, ETS, adenylates, and energy charge

**Fig. 5:**
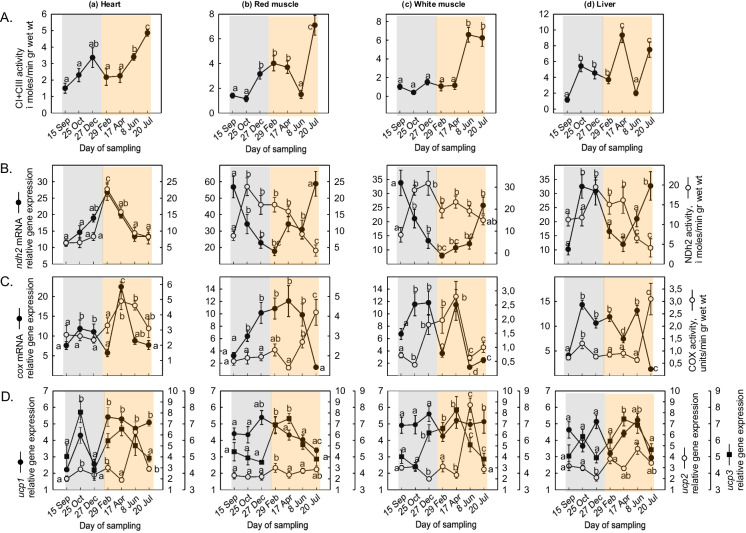
Seasonal variations in **A** C I + III activity, **B** *ndh*-2, **C** COX gene expression and enzymatic activity, and **D** *ucp*1, *ucp*2, and *ucp*3 gene expression levels in the (a) heart, (b) red muscle, (c) white muscle, and (d) liver of *Sparus aurata* (*n* = 6 preparations from different animals, values are presented as means ± S.D.). Light grey and light orange rectangles depict decreasing and increasing sea water temperature, respectively. Statistically significant differences (*p* < 0.05) between different samplings are indicated by lowercase letters

CI and CIII complex activity levels increased gradually in the heart from February to July (Fig. 5Aa). In the red muscle, CI and CIII complex activity levels increased from October to December, followed by a decline in June and increased again sharply in July (Fig. 5Ab). In the white muscle, the activity levels of CI and CIII complex increased from April to June (Fig. 5Ac), while in the liver, a peak was observed in April (Fig. 5Bd).

*ndh*-2 mRNA levels showed a gradual decrease in the heart from September to April but NDh-2 enzymatic activity levels increased from September to October (Fig. 5Ba). In the red muscle, *ndh*-2 gene expression levels exhibited a gradual increase from September to July, while NDh-2 enzymatic activity levels depicted a progressive decrease from October to July (Fig. 5Bb). *ndh*-2 mRNA levels in the white muscle did not show statistical changes and the enzymatic activity levels displayed a gradual increase from September to February (Fig. 5Bc). In the liver, a significant increase in *ndh*-2 mRNA levels was exhibited in June–July, while the enzymatic activity exhibited high values from September to December (Fig. 5Bd).

*cox* mRNA levels showed a sharp increase in the heart from September to April, while its enzymatic activity levels showed a significant increase from December to July (Fig. 5Ca). In the red muscle, both COX mRNA and enzymatic activity levels showed a similar pattern with the one observed in the heart (Fig. 5Cb). *cox* mRNA levels in the white muscle strongly decreased in April, while its enzymatic activity levels showed a gradual increase from February to June (Fig. 5 Cc). In the liver, *cox* mRNA levels displayed a significant increase from September to October followed by high expression values. However, COX enzymatic activity levels remained stable throughout the experimental period and only exhibited a sharp increase in July (Fig. 5Cd).

*ucp*1 and *ucp*3 mRNA levels in the heart significantly increased late in October, followed by a gradual decline from February to July. On the contrary, *ucp*2 mRNA levels peaked in June (Fig. 5Da). In the red muscle, *ucp*1 mRNA levels decreased from April to July, while *ucp*2 mRNA levels exhibited a significant increase in February. Additionally, *ucp*3 mRNA levels gradually increased in June (Fig. 5Db). In the white muscle, *ucp*1 mRNA levels decreased in February, whereas *ucp*3 mRNA levels gradually increased from October to April. *ucp*2 reached its peak mRNA levels in June (Fig. 5Dc). Overall, *ucp*1 mRNA levels displayed a steady decline from April to July, while *ucp*2 mRNA levels decreased in December. Meanwhile, *ucp*3 mRNA levels showed an increase in October, with a subsequent gradual increase from December to June (Fig. 5Dd).

**Fig. 6:**
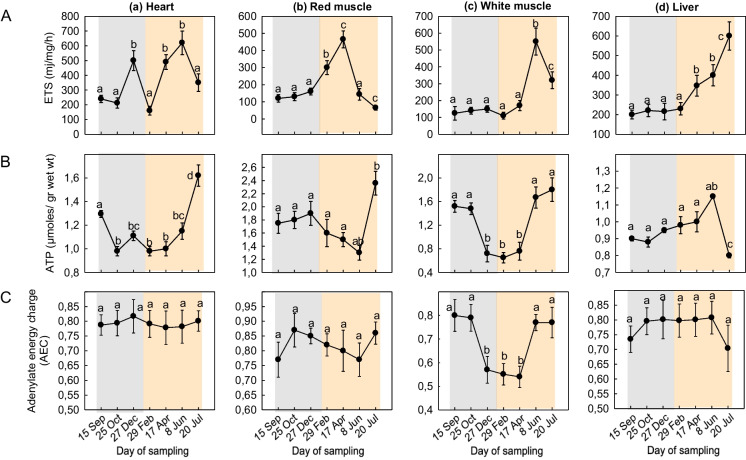
Seasonal variations in **A** ETS activity, **B** ATP, and **C** AEC levels in the (a) heart, (b) red muscle, (c) white muscle, and (d) liver of *Sparus aurata* (*n* = 6 preparations from different animals, values are presented as means ± S.D.). Light grey and light orange rectangles depict decreasing and increasing sea water temperature, respectively. Statistically significant differences (*p* < 0.05) between different samplings are indicated by lowercase letters

ETS levels exhibited a significant peak in the heart in December followed by a gradual increase from February to June (Fig. 6Aa). In the red muscle, ETS levels sharply increased from September to mid-April (Fig. 6Ab), while in the white muscle, ETS levels remained stable from September to April but intensely increased in June (Fig. 6Ac). In the liver, ETS levels showed a gradual increase from April to July (Fig. 6Ad).

ATP levels gradually increased in the heart from February to July (Fig. 6Ba). The red muscle exhibited a similar pattern compared to the one observed in the heart, but with a significant decline in June (Fig. 6Bb). The white muscle exhibited an increase in the summer months and specifically in June and July (Fig. 6Bc), while in the liver, no significant changes in ETS levels were observed (Fig. 6Bd).

AEC levels gradually increased from September to December in the heart (Fig. 6Ca). In the red muscle, AEC sharply increased from September to October followed by a gradual decline in June and an intense increase in July (Fig. 6Cb). AEC levels displayed a decrease in the white muscle from September to April, followed by an increase from April to July (Fig. 6Cc), while in the liver, AEC levels increased from September to October, remained stable until June, and sharply decreased in July (Fig. 6Cd).

### Indices of aerobic capacity and blood plasma glucose and triglycerides

**Fig. 7:**
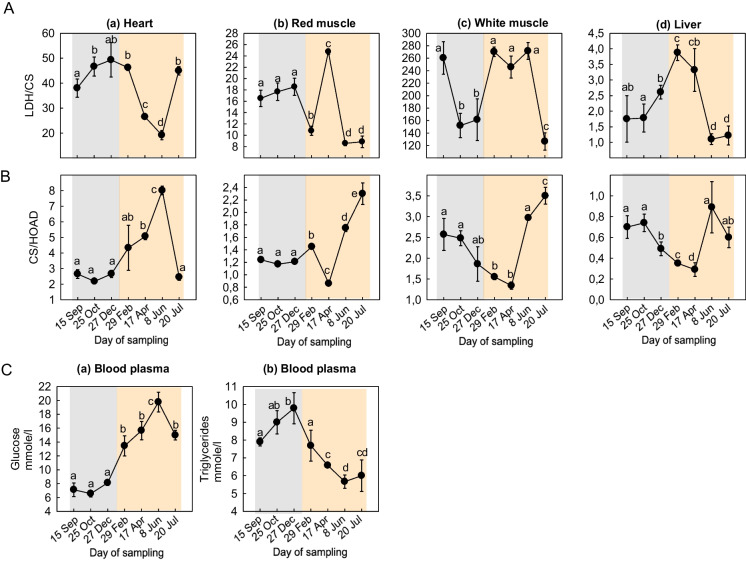
Seasonal changes in **A** LDH/CS; **B** CS/HOAD levels in the (a) heart, (b) red muscle, (c) white muscle, and (d) liver; and **C** blood plasma (a) glucose and (b) triglycerides levels of *Sparus aurata* (*n* = 6 preparations from different animals, values are presented as means ± S.D.). Light grey and light orange rectangles depict decreasing and increasing sea water temperature, respectively. Statistically significant differences (*p* < 0.05) between different samplings are indicated by lowercase letters

LDH/CS ratio in the heart progressively decreased in July (Fig. 7Aa), while in the red muscle, it sharply increased in mid-April, followed by a decrease until mid-July (Fig. 7Ab). However, in the white muscle, LDH/CS ratio increased from October until February and then again from April to June (Fig. 7Ac). In the liver, LDH/CS ratio exhibited a significant peak in April and thereafter decreased (Fig. 7Ad).

CS/HOAD ratio increased in the heart late in February (Fig. 7Ba). In the red muscle, CS/HOAD ratio decreased in April and then increased sharply in July (Fig. 7Bb). A similar pattern was observed in the white muscle (Fig. 7Bc). In the liver, CS/HOAD ratio gradually decreased from October to April, followed by a sharp increase in June (Fig. 7Bd).

Blood plasma glucose levels remained equal during decreasing sea water temperature and thereafter increased exhibiting the highest levels in June (Fig. 7Ca). On the contrary, blood triglycerides exhibited their highest levels during decreasing sea water temperature, and from February (when sea water temperature started increasing), these levels gradually decreased exhibiting the lowest values in June (Fig. 7Cb).

### Multivariate analysis

**Fig. 8:**
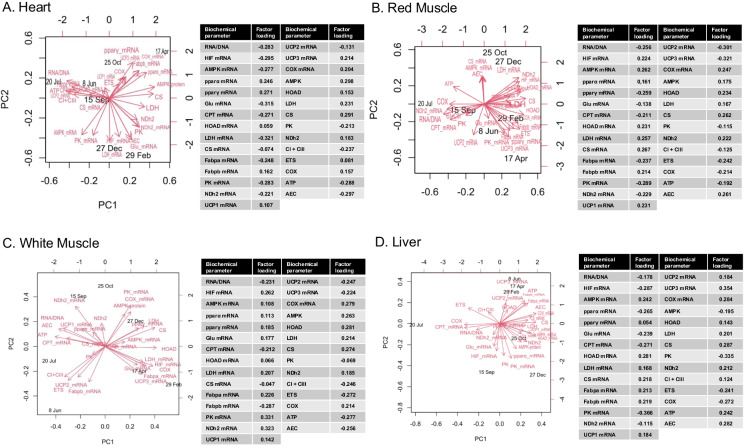
Correlations of biochemical responses in the heart (**A**), red (**B**) and white muscle (**C**), and liver (**D**), of *Sparus aurata* with each of the first two principal components (PCs) in the multivariate analysis (PCA construction predictors are depicted as red vector arrows)

In the heart, *pparα* mRNA, AMPK, LDH, CS, NDh2, and AEC were positively correlated with PC1; RNA/DNA, *cpt* mRNA, *fabpa* mRNA, *ucp*2 mRNA, CI + CIII, and ATP were negatively correlated with PC1; *pparγ* mRNA, *fabpb* mRNA, *ucp*1 mRNA, *ucp*3 mRNA, *cox* mRNA, HOAD, and COX were positively correlated with PC2; and finally, *hif* mRNA, *ampk* mRNA, *glu* mRNA, *ldh *mRNA, *pk* mRNA, *ndh2* mRNA, PK, and ETS were negatively correlated with PC2. Overall, 36.45% of the variance was attributed to PC1, while 27.02% was attributed to PC2. Cumulatively, PC1 and PC2 explain 63.47% of the total variance in the dataset (Fig. 8A).

In the red muscle, *hif* mRNA, *pparα* mRNA, *hoad* mRNA, *fabpb* mRNA, *ucp*1 mRNA, *cox* mRNA, AMPK, HOAD, LDH, CS, and NDh2 were positively correlated with PC1; RNA/DNA, *cpt* mRNA, *ndh2* mRNA, PK, CI + CIII, COX, and ATP were negatively correlated with PC1. Regarding PC2, *ampk* mRNA, *ldh* mRNA, cs mRNA, and AEC were negatively correlated with PC2, and *pparγ* mRNA, *glu* mRNA, *fabpa* mRNA, *pk *mRNA, *ucp*2 mRNA, *ucp*3 mRNA, and ETS were negatively correlated with PC2. 41.42% of the variance was attributed to PC1, while 25.54% was attributed to PC2. Cumulatively, PC1 and PC2 explain 63.47% of the total variance in the dataset (Fig. 8B).

In the white muscle, *hif *mRNA, *ampk *mRNA,* pparγ* mRNA, *glu* mRNA, *hoad* mRNA, *ldh* mRNA, *fabpa* mRNA, HOAD, LDH, CS, and COX were positively correlated with PC1; RNA/DNA, *cpt* mRNA, ATP, and AEC were negatively correlated with PC1; *pparα* mRNA, *pk* mRNA, *ndh2 *mRNA, *ucp*1 mRNA, *cox* mRNA, AMPK, and NDh2 were positively correlated with PC2; and *fabpb *mRNA, *ucp*2 mRNA, *ucp*3 mRNA, CI + CIII, and ETS were negatively correlated with PC2. 30.22% of the variance was attributed to PC1, while 29.09% was attributed to PC2. Cumulatively, PC1 and PC2 explain 59.31% of the total variance in the dataset (Fig. 8C).

In the liver, *ampk* mRNA, *pparγ* mRNA, *hoad* mRNA, *ldh* mRNA, *cs* mRNA, *fabpb* mRNA, *ucp*1 mRNA, *cox* mRNA, LDH, CS, NDh2, and AEC were positively correlated with PC1; RNA/DNA, *cpt *mRNA, ETS, and COX were negatively correlated with PC1; *fabpa* mRNA, *ucp*2 mRNA, *ucp*3 mRNA, HOAD, CI + CIII, and ATP were positively correlated with PC2; and *hif* mRNA, *pparα* mRNA, *glu* mRNA, *pk*mRNA, *ndh2* mRNA, AMPK, and PK were negatively correlated with PC2. Overall, 29.32% of the variance was attributed to PC1, while 29.18% was attributed to PC2. Cumulatively, PC1 and PC2 explain 58.5% of the total variance in the dataset (Fig. 8D).

## Discussion

The Mediterranean Sea is considered a hot spot for marine life, hosting inter alia, numerous species of high economic value and importance for fisheries and aquaculture. At the same time, it is threatened by climate change effects with temperature extremes, often exceeding aquatic species lethal limits. It should be hence noted, that although extremely high seawater temperatures are recorded during summer months, winter seawater temperatures are still quite low, particularly close to the surface where fish mariculture takes place. As a result, cultured fish are exposed to both low and high temperature extremes that even in the cases that mortalities are avoided due to practices carried out by the farmers, they are facing intense cold and heat stress, decreased energy consumption and metabolism, and eventually decreased productivity. To explore these phenomena in one of the major representatives of Mediterranean aquaculture, i.e., *S. aurata*, a large number of biomarkers were measured in a seasonal basis, including RNA/DNA that could give a general image for the protein synthesis and together with AMPK can provide insights for the energy status and homeostasis. To accompany homeostasis, Glu, PK, L-LDH, CS, NDH-2, and COX1 expression, together with ATP, AEC and ETS, and blood glucose can enlighten the metabolic status of the fish, whereas Hif-1a expression is related with oxygen absorbing situations such as hypoxia or normoxia that could be the result of abnormal temperature. Additionally, CPT, HOAD, PPARα/γ, Fabp2a/b, and blood triglycerides can provide useful info concerning lipid metabolism and fatty acids and therefore enlighten productivity of the fish. Overall, our results reveal various adaptive mechanisms to seasonal environmental fluctuations acclimatization, discussed in detail below, separately for winter and summer temperatures.

### Metabolic status

RNA/DNA ratio data suggested decreased protein synthesis, which may reflect a decreased feeding ratio during the cold acclimatization period. Nevertheless, this biomarker is too generic to clearly define solely the nutritional condition of an aquatic species, let alone in the field. Hence, the decreased protein biosynthesis indicated by RNA/DNA ratio may probably reflect the decreased physiological performance in terms of metabolic rate, in a more generic point of view. In other words, low temperature might have directly caused the decreased protein synthesis, regarding the production of various other proteins, potentially related to cold stress. Farmed *S. aurata* exhibits a lethal limit below 5 °C (Ravagnan 1978), while at temperatures below 15 °C fish activity, growth rates are significantly decreased. The latter is documented by several studies which have shown decreased feeding rates in fish during winter months (Tort and Rotilland 1998; Tort et al. 1998; Sarusic 1999; Ibarz et al. 2003, 2007a).

Acclimatization to increasing temperatures in fish, on the other hand, is crucial for coping with thermal tolerance and avoiding hypoxia (Pörtner et al. 2007; Pörtner 2012). A temperature increase was recorded from February to April, however peaking during the summer months. Notably, RNA/DNA ratio showed a significant increase in all examined tissues, suggesting enhanced growth rates and protein biosynthesis during warm acclimatization (Tanaka et al. 2008; Foley et al. 2016). This increase is not only related with feed intake and is in line with some particular genes examined, as described below.

Conversely, the observed *hif-*1α expression increase in all examined tissues during colder periods probably does not necessarily reflect systemic hypoxic conditions, as in other fish species undergoing cold acclimatization (Katschinski et al. 2002; Rissanen et al. 2006; Feidantsis et al. 2015). Although *hif-*1α expression is also observed in normoxia, the low water temperature may contribute to the formation of more complex responses in fish. Notably, in colder periods, decreased Hif-1α would be expected, mainly on account of dissolved oxygen content and the decrease of metabolism, a fact that was not the case here. Instead, probably the increase in *hif*-1α during winter indicates that on the one hand this biomarker is not on its own sufficient to discriminate hypoxic from normoxic conditions and on the other hand enhances the role of HIF-1 as one of the primary genes involved in the homeostatic process (Ziello et al. 2007). Under this scenario, *hif*-1α increase may be due to non-proper transfer of oxygen to mitochondria as a result of homeostasis regulation. Interestingly, while AMPK protein levels in the heart showed decreased levels, *ampk* gene exhibited high mRNA expression levels, suggesting a complex regulatory mechanism. This finding indicates a preparatory mechanism that may be eventually omitted since temperature fluctuations were not continued. From a metabolic point of view, this outcome reflects the reduction in feed intake, in line with the scenario of winter syndrome. A similar pattern was also observed in the liver. In the red muscle, however, an opposite pattern was observed, indicating a high metabolic capacity at decreasing temperatures. Previous studies have demonstrated the involvement of the Hif-1α transcriptional pathway on metabolic pathways gene expression, energy consumption, and the function of both heart and skeletal muscle in mammals (Mason et al. 2004; Huang et al. 2004; Rissanen et al. 2006). Notably, a study by Nie et al. (2020) observed an increase in *ampk* gene expression in olive flounder *Paralichthys olivaceus* (Temminck & Schlegel, 1846) exposed to low temperatures.

### Carbohydrate and lipid metabolism

Fish typically metabolize glucose slower at lower temperatures, and subsequently, they rely less on carbohydrate metabolism. The February increase observed in *glu* gene expression in the heart suggests an adaptive physiological response in response to cold, since increased glucose levels are known to enhance *glu* expression (Wang et al. 2020).

Concomitantly, the increases in *ldh* and *pk* gene expression, along with their elevated enzymatic activity in the heart during low sea water temperatures (December-February), underscore the metabolic adaptations that take place. Our results also indicated a decline in ATP levels across all tissues during cold acclimatization, suggesting the supply of energy through less energy-efficient anaerobic metabolism in the heart, since hypoxia starts to set in. This is supported by the increase in *hif*-1α gene expression in the cold acclimatized *S. aurata*, which likely promotes the expression of glycolytic genes, such as *ldh*, while mobilizing lipid metabolism. Hif-1α activation in parallel with increased glycolytic gene expression has been observed in both heart and gills of the crucian carp *Carassius carassius* (Linnaeus, 1758) (Gracey et al. 2004). In the same species, Rissanen et al. (2006) reported a transcriptional response of Hif-1α in the heart during cold acclimatization. Lactate, produced under hypoxic conditions in the heart (Gamperl and Driedzic 2009; Lague et al. 2012; Becker et al. 2013), highlights the reliance of several fish species on anaerobic metabolism during low-temperature environments to sustain energy requirements (Gamperl and Driedzic 2009; Clow et al. 2017). It has been proposed that reduced oxygen levels result in decreased ATP concentrations and elevated lactate production (Currie et al. 1999). Taken altogether, cold exposure in fish is associated with significant depletion of glycogen reserves, indicating a decreased reliance on carbohydrate-based energy production (Larsen et al. 2001; Driedzic and Short 2007).

L-LDH and PK enzymatic activities in the red muscle exhibited significant increases in April, indicating a rise of glycolysis and overall metabolic capacity, consistent with findings in various fish species (Iftikar and Hickey 2013; Iftikar et al. 2014). During the same period, AMPK showed both transcriptional and translational responses at elevated temperatures. AMPK plays a pivotal role in stress response, enabling organisms to adapt to fluctuating environmental factors, including temperature and hypoxia (Wang et al. 2012). Ekström et al. (2017) reported diminished substrate oxidation capacity in perch *Perca fluviatilis* (Linnaeus, 1758) at high temperatures, a fact which prompts increased anaerobic metabolism for ATP generation due to hypoxia (Pörtner 2004).

The white muscle serves as the primary anaerobic muscle (Hochachka and Somero 2002). A marked increase in L-LDH activity in mid-April, followed by a rise in PK activity late in July, supports the notion that muscle resorts to glycolysis for energy maintenance at warm acclimatization (Hochachka and Somero 2002). Interestingly, L-LDH and PK enzymatic activities are typically higher in Antarctic species compared to those from northern temperate zones (Crockett and Sidell 1990), and lower activity of these enzymes indicates hypoxia resistance in the heart (West et al. 2011). Concurrently, a decrease in Glu gene expression highlighted a shift towards anaerobic glucose production in the present study.

In the pearl cichlid *Geophagus brasiliensis* (Quoy and Gaimard 1824), an increase in glycogen content coupled with decreased L-LDH activity in the liver suggested a metabolic blockade to oxidation (Pereira et al. 2018). The present results exhibited a gradual decline of CS enzymatic activity in the heart, red muscle, and white muscle in the summer months. A significant decrease in CS activity implies metabolic suppression under hypoxic conditions, a fact which eventually leads to the activation of anaerobic metabolism (Pereira et al. 2018). The decreasing CS enzymatic activity during this period may reflect the initiation of mitochondrial dysfunction, which may be enhanced under regimes of continuously increasing temperatures, although warm acclimatization results to an increase in the mitochondrial capacity of the gilthead seabream. The latter is also supported by the higher at elevated temperatures LDH/CS ratio which highlights the transition from aerobic to anaerobic metabolism, emphasizing the physiological trade-offs which enhance survival in dynamic habitats. The decline in CS enzymatic activity suggests decreased mitochondrial capacity in response to warm acclimatization (Iftikar et al. 2014), while increased HOAD activity facilitates fatty acid oxidation for energy production (Ekström et al. 2017). The latter agrees with the results of the present study in which the transcriptional responses of HOAD, a sharp increase in CPT1 A and a decrease in PPARs, indicated lipid transport and utilization for β-oxidation in the tissues of *S. aurata*.

The higher at elevated temperatures LDH/CS ratio further supports the transition from aerobic to anaerobic metabolism, as demonstrated by increased LDH levels and LDH/CS ratios in sea bream larvae (Michaelidis et al. 2007; Pimentel et al. 2020). The increased L-LDH activity and LDH/CS ratios in sea bream larvae at high temperatures indicate a metabolic shift in response to energy demands amid ocean acidification (Pimentel et al. 2020). Variability in L-LDH/CS ratio between tissues serves as a relative indicator of adjustments across anaerobic and aerobic metabolism, with lower values signifying heightened oxidative activity (Hochachka et al. 1983). Our results suggest a preference for carbohydrate oxidation at elevated temperatures. A decrease in both HOAD activity and CPT1 A transporter gene expression indicates that carbohydrates serve as the primary energy substrate in the liver. Conversely, the sharp increase in CPT1 A expression and the decline in PPARs from April to July reflect lipid mobilization for energy production, activation of metabolism, and increased food intake rates.

Notably, the elevated enzymatic activity of HOAD in the heart, white muscle, and liver suggests increased energy demands during decreasing temperatures. It has been shown that lipid absorption in the liver can occur rapidly to cover immediate energy demands, responding within a few days to changes in fish metabolic needs (Ibarz et al. 2007b). While these results are contradictory with the ones of other studies such as the one of Pelusio et al. (2021), it should be highlighted that the afore mentioned study is a laboratory one and therefore does not reflect the complexity of field studies. Although both lipid and carbohydrate metabolism seem to be activated in cold acclimatized fish, the present results indicate that lipid metabolism is indeed mostly favored at decreasing temperatures (as also seen by the increased blood plasma triglyceride levels), while carbohydrate metabolism is activated at higher temperatures (as also seen by the increased blood plasma glucose levels) in Mediterranean fish such as *S. aurata*. It has been shown however that fish can incorporate carbohydrates, such as lactate, into total lipids (Jayasundara and Somero 2013). Specifically, in physiological states requiring increased energy (e.g., fasting or stress), stored triacylglycerols in perivascular fat, liver, and muscle are catabolized into glycerol and fatty acids (Sheridan 1988).

The expression of genes such as *cpt*1 A, *ppar*α, and *ppar*γ indicates lipid mobilization and transport for β-oxidation to fulfill energy requirements. PPARs regulate CPT enzymes, which are crucial for fatty acid oxidation at decreasing temperatures (Kersten et al. 1999; Leone et al. 1999; Song et al. 2010; Liu et al. 2006). Several studies have demonstrated increased PPARα expression in the liver and intestine during starvation periods (Kersten et al. 1999; Leone et al. 1999; Leaver et al. 2005). Additionally, during starvation, a decrease in malonyl-CoA levels facilitates energy production through fatty acid oxidation, while at the same time, this decrease activates CPT1 A (Robinson and Zammit 1982; Liang 2023). The observed, in the present study, increase in PPARα expression in the liver, heart, and red muscle of *S. aurata* aligns with findings from other studies highlighting similar responses during starvation (Forman et al. 1997; Leaver et al. 2005).

Interestingly, FABPs are related to PPARs, and both are known to increase in response to stress (Schachtrup et al. 2004). According to mRNA levels estimated, expression of *fabp*2a/b in the heart, white muscle, and liver were elevated during cold exposure, suggesting lipid accumulation at decreasing temperatures. Several studies demonstrated similar expression patterns of Fabp2a/b and PPARα/γ proposing the hypothesis that Fabp expression is involved in PPAR signaling (Cocci et al. 2015; Palermo et al. 2016). Nevertheless, since in our study only gene expression was analyzed at mRNA levels, further molecular approaches are needed to confirm this pathway in fish.

### Mitochondrial metabolism and Krebs cycle

In general, both *ucp*1 and *ucp*3 genes exhibited increased expression in the heart, while only *ucp*3 expression levels increased in the white muscle during cold acclimatization. Notably, *ucp*1 expression increased late in October in the liver. UCP expression has been linked with thermal resistance in various fish species (Hilton et al. 2010). In the context of thermal resistance, both UCP1 and UCP3 enact a critical role to protect mitochondria from lipid-induced damage (Schrauwen and Hesselink 2004). Bermejo-Nogales et al. (2010) observed high UCP3 expression levels in the white muscle and lower levels in the heart and red muscle of *S. aurata* during winter adaptive cold response. Reduced feeding rates are associated with upregulated UCP3 expression, as demonstrated in common carp *Cyprinus carpio* (Linnaeus, 1758) during starvation (Jastroch et al. 2005). An increase in UCP3 expression often occurs when energy demands exceed energy supplies, linking UCP3 to lipid metabolism (Nabben and Hoeks 2008).

Additionally, increased UCP3 expression enhances oxidative phosphorylation efficiency, yielding positive outcomes in the heart (Bo et al. 2008) and skeletal muscle (Jiang et al. 2009) as an antioxidant defense against cold induced oxidative stress. Likewise, in zebrafish *Danio rerio* (F. Hamilton, 1822), an upregulation of UCP3 was observed in the brain alongside with increased Hif-1α expression levels in response to hypoxic cold conditions (Tseng et al. 2011). It should be highlighted that decreased food intake during cold acclimatization is correlated with increased oxidative capacity in *S. aurata* (Bermejo-Nogales et al. 2010).

The hypoxic environment created during low-temperature periods adversely affects aerobic oxidation capacity. Although *hif-1a* expression was only estimated at mRNA levels, without obtaining information concerning post expression mRNA degradation, we assume that this is not clearly attributed to hypoxia, during which this gene is also expressed, but also to homeostasis regulation owing to cold stress and potentially winter syndrome. In line with this assumption, oxygen- and capacity-limited thermal tolerance (OCLTT) hypothesis, fish exhibit significantly decreased aerobic capacity at low temperatures (Pörtner 2012). One key indicator of aerobic mitochondrial function is CS. In the present study, the expression of *cs* gene and its enzymatic activity displayed a synchronized pattern, suggesting an overall compensatory response to decreasing temperature conditions. However, increased mRNA levels of various enzymes without a corresponding rise in enzyme activity can be interpreted as a preparatory mechanism against unfavorable conditions. Nevertheless, the translational process becomes more active and synchronized to mRNA production under intensified stress (Somero 2010, 2020).

The present results highlight tissue-specific differences which are related to tissue-specific energy needs. The activities of mitochondrial complex I (NDH-2) and IV (COX) demonstrated similar trends. In the heart and red muscle, *cox* gene expression and its enzymatic activity concurrently increased, thus reflecting parallel transcriptional and translational processes. However, these mitochondrial complexes’ activation seems to be species-specific. While in certain temperate species, *cox* gene expression is unaffected by temperature changes (Breyer and Moyes 2011), the latter does not apply to other fish species. Specifically, while zebrafish were not significantly affected by temperature changes, common dace *Leuciscus leuciscus* (Linnaeus, 1758) and goldfish *Carassius auratus* (Linnaeus, 1758) exhibited increased COX expression under cold conditions (Duggan et al. 2011). Notably, in cold-exposed cod *Gadus morhua* (Linnaeus, 1758), both *cox* and *cs* gene expression increased in the white muscle. Furthermore, the COX activity in cod liver was found to be higher in populations from colder environments (Lucassen et al. 2003). These alterations in COX and CS enzyme activities in relation to temperature suggest enhanced aerobic capacity in the red muscle under decreasing temperature conditions, corroborating findings from previous studies (Pörtner 2002; 2004; Ibarz et al. 2010).

The observed decline in COX and ETS activity in the herein examined tissues suggests decreased respiration rates during warm acclimatization. While temperature increase leads to enhanced oxygen consumption, Krebs cycle and oxidative phosphorylation reactions also become affected, enhancing mitochondrial membrane fluidity and elevating proton rates (Pichaud et al. 2019). Gracey et al. (2004) found higher NDH-2 expression in fish exposed to cold compared to those which were warm acclimatized, suggesting NDH-2’s role as a gatekeeper in the respiratory chain during oxygen-related stress (Gospodaryov et al. 2020). Despite decreases in COX, CS, and NDH-2 enzymatic activities, *ucp*2 and ETS exhibited increased expression in the heart, white muscle, and liver by mid-June, indicating a rise in proton pumping at increasing temperatures. Furthermore, CI and CIII complexes increased during warm acclimatization in all tissues, suggesting enhanced respiration rates and proton transfer. In the Antarctic eelpout *Pachycara brachycephalum* (Pappenheim, 1912), UCP2 expression was found to increase during warm acclimatization, indicative of enhanced mitochondrial proton pumping (Mark et al. 2006) and increased mitochondrial capacity (Pörtner et al. 2007, 2022).

ATP concentrations increased in the heart, red muscle, and white muscle during warm acclimatization, supporting thus high energy charge levels in *S. aurata* examined in the present study. Tissues with higher mitochondrial contents typically maintain elevated adenylate loads and minimize ADP, AMP, and inorganic phosphate levels to reduce glycolytic stimulation, thus favoring the utilization of carbohydrate-free substrates (Holloszy and Coyle 1984). However, metabolic processes are sensitive to temperature changes; metabolic rates decrease due to reduced feeding, resulting in lower ATP levels (Jobling 1993). Aerobic ATP production is confined to oxidative muscles as temperatures fall (Egginton and Sidell 1989; Pörtner 2002, 2004; Guderley and Pörtner 2010; Feidantsis et al. 2018).

Overall, our study indicated an aquaculture adaptation and physiological performance of the gilthead seabream in sea surface cage farms that constitute the intensive mariculture system in the Mediterranean Sea. While *S. aurata* wild populations are benthic, it seems that during the last 4–5 decades when this farming system takes place, this species populations have been physiologically adapted to this new environment. It should be however noted that we analyzed a large number of biomarkers, some of which were only estimated on mRNA level, without protein measurement, with this being a limitation of our study. Additionally, although some important physicochemical parameters were recorded and correlated with physiological results, field studies possess the drawback of not having the possibility to evaluate all parameters that may influence the biological parameters of a fish.

## Conclusion

**Fig. 9:**
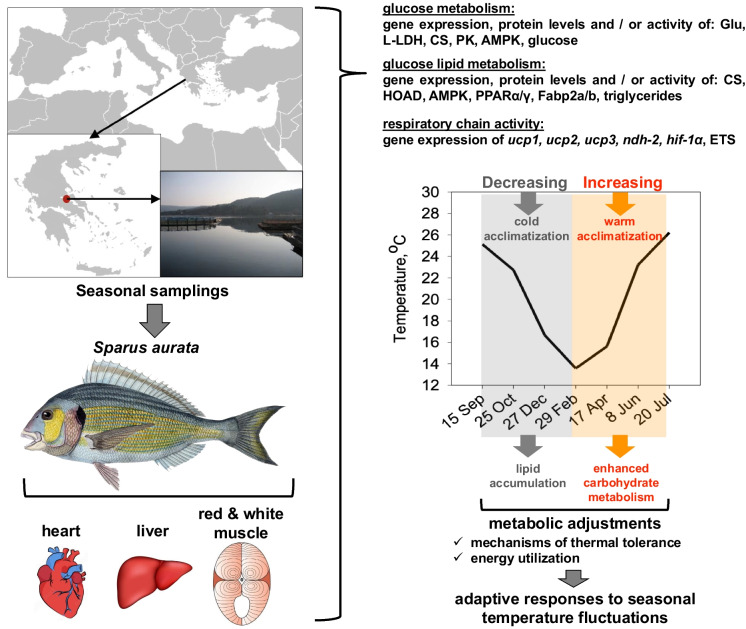
Summarized model of *S. aurata* seasonal metabolic responses to cold and warm acclimatization

Metabolic patterns of *S. aurata* highlight the complex physiological and biochemical adaptations that occur in response to seasonal temperature changes. The study revealed different metabolic responses which were distinctly divided in two periods, as also seen by the PCA results: seasonal cold and warm acclimatization, as graphically visualized in Fig. 9. During colder months, decreased protein synthesis aligned with studies which indicate diminished feeding rates in fish during winter. The upregulation of *hif*-1a during these periods additionally indicated a hypoxic response, crucial for energy production and metabolic regulation under cold induced internal hypoxic conditions. Conversely, acclimatization to warmer temperatures resulted in increased metabolic capacity, as indicated by the significant rise in the RNA/DNA ratio and metabolic enzymatic activities. Although, in liver, this biomarker was increased in October and December, this increase does not imply a subsequent increase in metabolism, not only because of the lower levels, but also on account of the coldest temperature that was observed later, in February, when RNA/DNA was at lowest level. Our findings, thus, support the notion that gilthead sea bream enhances its aerobic capacity and metabolic efficiency during warmer months. It should be underlined that the differences in transcriptional and post-translational levels of the examined biochemical parameters can be attributed to the fact that mRNA levels may increase quickly and then decrease rapidly, whereas the levels of the corresponding protein may rise slowly and persist for a much longer period than mRNA levels (Buckley and Klaassen 2007). Additionally, protein turnover, post-translational modification, alternative splicing, translational efficiency, and other processes rates may act independently of transcription to alter the proteome (Vogel and Marcotte 2012). Ultimately, these findings contribute with valuable insights into the thermal tolerance and metabolic strategies of *S. aurata*, strengthening our knowledge concerning the impact of climate change on fish physiology and ecology in the future, as well as aquaculture productivity at a seasonal level. Future research should focus on molecular mechanisms underlying these adaptations in other fish species and examine the broader ecological implications of thermal stress in marine environments.

## Data Availability

No datasets were generated or analysed during the current study.
